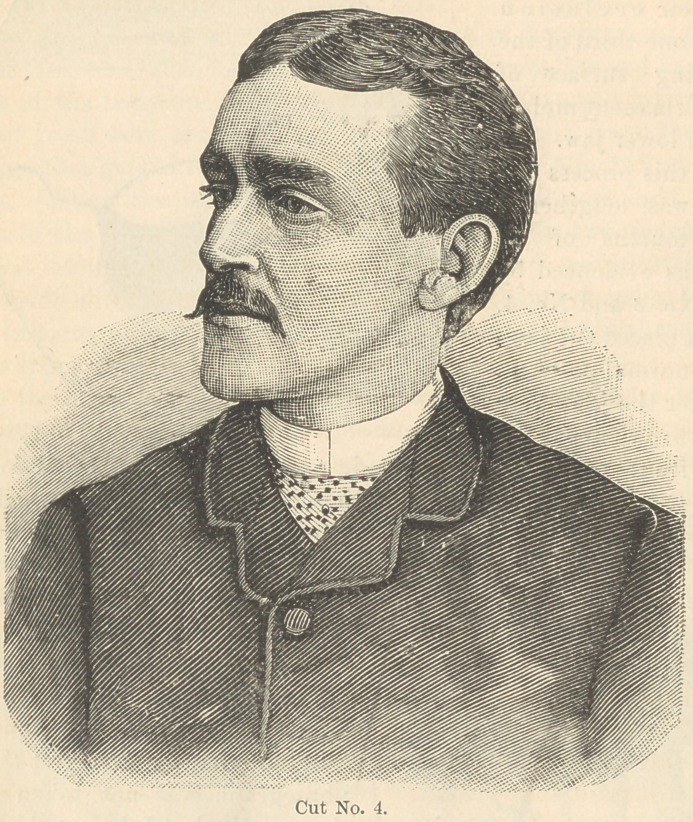# American Medical Association

**Published:** 1884-12

**Authors:** Jno. S. Marshall


### AMERICAN MEDICAL ASSOCIATION.

#### SECTION OF DENTAL AND ORAL SURGERY.

REPORTED FOR THE INDEPENDENT PRACTITIONER BY JNO. S. MARSHALL, M. D.

"Report of a peculiar case of malformation of the jaws, and the treatment;"* by W. W. Allport, M. D., D. D. S.

*Mr. Chairman and Gentlemen:*

The plaster casts I exhibit to you are models of a case that I have recently had under treatment. It had excited considerable interest and discussion among some of the surgeons and dentists of his native State, but no definite treatment had been decided upon.

On taking up his residence in Chicago, the dentists at his home in New Hampshire advised that he put himself into my hands for such treatment as might be thought best, and the case is, I think, of sufficient interest to warrant me in reporting it to the section.

Mr. F. L. S——, aged twenty-five years, consulted me January 22, 1884, for a malformed condition of both maxillary arches. The young gentleman is a graduate of Harvard College, and a civil engineer by profession. The malformation was such as to make it

* Journal of the American Medical Association, Oct. 25, 1884.—Through the kindness of Dr. N. S. Davis, the editor, we are able to print the cuts with the paper.

impossible for him to properly masticate his food, and his personal appearance, especially when eating, was very unsightly, and a source of great mortification.

The trouble was largely due to a lack of development of the upper jaw, consequent upon a failure in the formation and development of the temporary and permanent teeth. Just how many temporary teeth were erupted I am unable to say, but from the best information gained it is evident he did not have the full complement. When the examination was made there were only seven teeth in the superior jaw, two temporary cuspids, two permanent central incisors, and three molars; the first and second upon the right side and the second upon the left, all of them somewhat imperfect in form, and these are the only permanent teeth that have made their appearance in this jaw.

In the lower jaw the six anterior temporary teeth were still in position, though much worn away on the cutting edges (see cut No. 2), and the second temporary molar on the left side, and both temporary molars on the right side, each considerably decayed. The permanent teeth were the first bicuspids on the left side, and the first molars on either side.

Cut No. 1.

The angle of the lower jaw was less acute than normal, causing the jaw to protrude to a slight extent, but this would have caused no marked deformity had the superior jaw been properly developed.

Cut No. 1 represents a front view of the case (taken from plaster casts), with the jaws closed to the full extent.

Cut No. 2 represents a side view of the same models, which shows the extent of the recession of the upper jaw and the protrusion of the lower; also that the occlusion was such that when the jaws were closed the bite was so short as to produce a most unsightly appearance of the face. To lengthen the bite permanently, gold crowns were fitted over the only superior molar of the left side, and so shaped as to cover in their occlusion about one-third of the grinding surface of the permanent molars in the lower jaw.

Cut No. 2.

By this process the bite was lengthened three-fourths of an inch, as evidenced by cuts No. 3 and No. 4, which placed the jaws in a normal position.

After the jaws were thus thrown apart the four front teeth were extracted, a gold plate was fitted to the mouth and secured by heavy clasps around the gold crowns, and then mounted with artificial teeth, secured to the plate by rubber attachments, as represented in cut No. 3. Cut No. 4 is taken from a photograph of the gentleman after the operation was completed.

Cut No. 3.

It is a matter of regret that a photograph was not taken with the mouth closed, before the jaws were permanently thrown apart by the gold crowns, that the great change in the expression might be seen and contrasted with cut No. 4. But this can readily be imagined.

DISCUSSION.

*Dr. Friedrich*—Said he believed he had seen a case reported which, he thought, had been treated somewhat after the manner described by Dr. Allport, though in that case the teeth were not extracted, but the plate was made to fit over them.

Cut No. 4.

*Dr. Williams*—Moved that, owing to the lateness of the hour and the desire of many to leave by the early evening trains, discussion of the paper be passed.

The section then adjourned.

---

### PROGRESS UNDER DIFFICULTIES.

A dental journal, to be called the *Dental Review,* is to be established in Russia. What can be its scientific status in a country in which, by an imperial decree, the works of Agassiz, Huxley, Lubbock, Herbert Spencer, Vogt, Zimmerman, and Charles Darwin, are under interdict; a land in which a demonstrated scientific fact is not truth unless it receives the approval of the church through its head?

## Figures and Tables

**Cut No. 1. f1:**
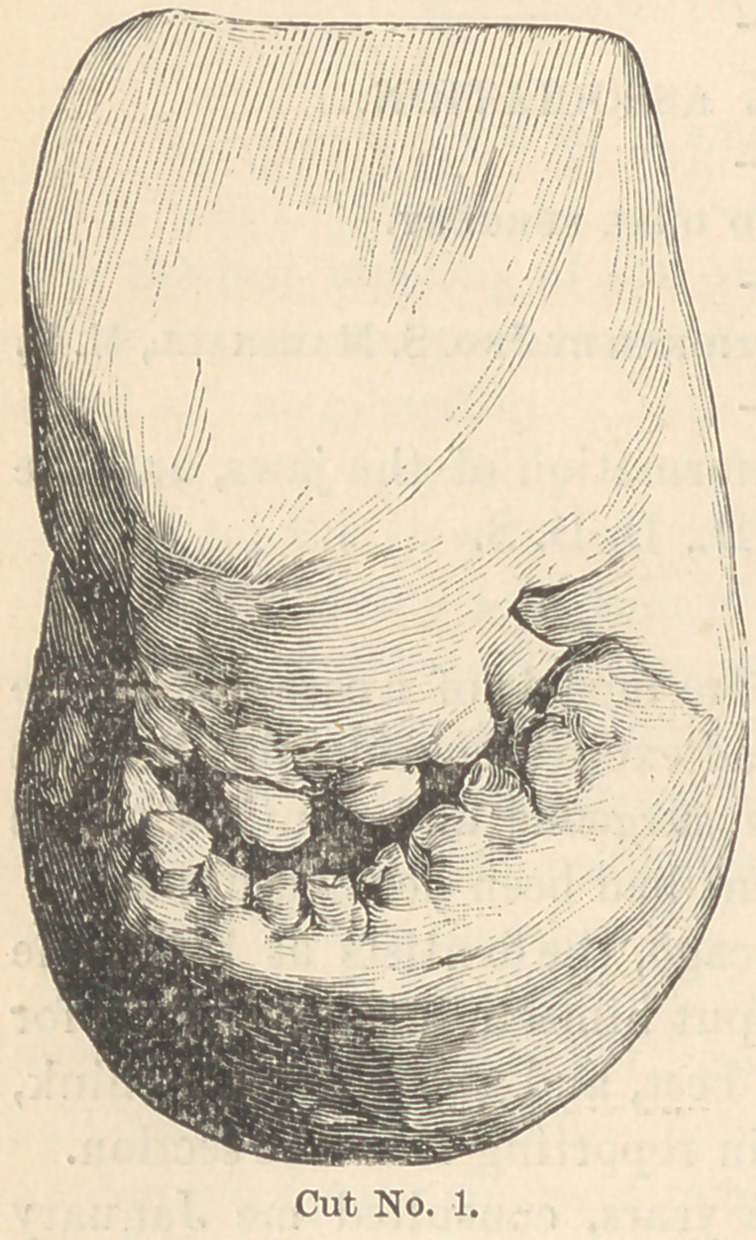


**Cut No. 2. f2:**
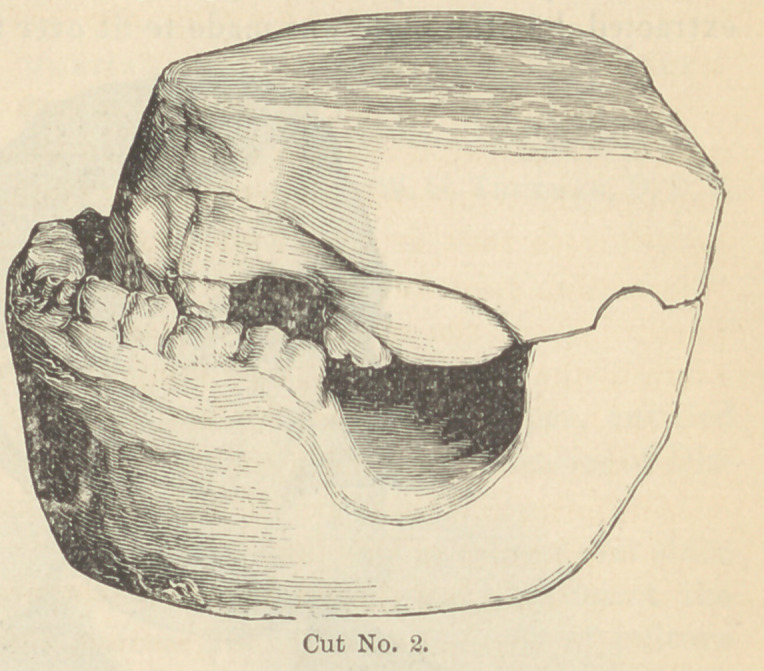


**Cut No. 3. f3:**
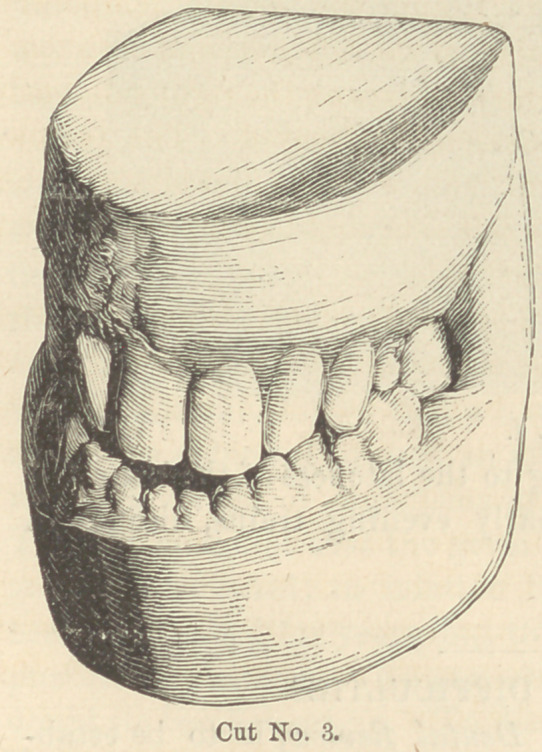


**Cut No. 4. f4:**